# Nursing Perspectives: Reflecting History and Informal Coercion in De-escalation Strategies

**DOI:** 10.3389/fpsyt.2019.00231

**Published:** 2019-04-24

**Authors:** Franziska Rabenschlag, Christoph Cassidy, Regine Steinauer

**Affiliations:** ^1^Department of Nursing Development and Research, University of Basel Psychiatric Clinics (UPK), Basel, Switzerland; ^2^Department of Psychiatric Services of the Canton of Aargau (PDAG), Windisch, Switzerland

**Keywords:** informal coercion, de-escalation, nursing, coercion history, nursing practice

## Abstract

Health professionals like nurses respond to aggression and violence with de-escalation techniques, and still often with coercive measures. Such measures applied by institutions are often rooted in historically grown traditions rather than evidence, reflection, or formation. In this article, we present de-escalation strategies integrating a high and critical awareness toward traditions and the practice of formal and informal coercion.

## Introduction

Health professionals may respond to aggression with de-escalation techniques, but a still predominant response to aggression and especially violence in psychiatric settings is a “physical” one. We have to think about which uses of measures in which situations of everyday life are really necessary, or could be replaced by other creative ideas or practices. Within these perspectives, we focus on a nursing approach, being aware that aggressive behavior affects the whole treatment team in mental health settings.

### Brief Historical Overview and Formal and Informal Coercion

In constitutional countries, mentally ill people are the only human beings who can be detained without being accused of an offense. Compulsory measures are often based on aggressive behavior aiming to calm down a situation and the involved persons (1). Its use, though based on judicial and ethical principles or guidelines (see, for example, DGPPN 2018), negatively impacts attitude toward treatment and is always perceived as negative by patients (2). Patients reject compulsory measures more distinctly than health professionals and even more clearly if they have already experienced those measures and if they were admitted involuntarily (3). The most strongly rejected form of coercive measures for patients, relatives, and health professionals are net beds (which are not used any more in Switzerland), fixations, and seclusion (3). The use of either fixation or seclusion or both in psychiatric institutions is often determined by regional history and traditions of institutions and management and is currently being questioned in many European countries. Empirical evidence or definable indicators of the benefit or harm of applying coercive measures are rare. Psychiatric institutions with a psychiatric care contract are very often trapped between help and violence, the expectations of authorities and the public, as well as the expectations of the patients and their families or relatives.

It is not only formal coercion but also informal coercion that is looked upon as negative and often hampers a therapeutic relationship (4, 5). Informal coercion is common but underestimated by health professionals (6). Informal coercion or treatment pressure (7) comprises subtle forms of communication mostly with the aim of preventing formal coercive measures or of improving treatment adherence (8). It can range from persuasion or inducement to more distinct forms like threats (9). Szmukler and Appelbaum (10) divided informal coercion into hierarchical degrees of persuasion, interpersonal leverage, inducement, and threats. A study from 1998 (11) also revealed the demonstration of force as a relevant form of coercion. The authors grouped the forms of coercion into nine degrees: persuasion, inducement, threats, show of force, physical force, legal force, request for a dispositional preference, giving orders, and deception (11).

The important fact is the underestimation in particular of stronger forms of informal coercion and formal coercion (6, 7). Yet, health professionals with a positive attitude toward weaker forms of informal coercion, like persuasion or leverage, tend to underestimate its occurrence more than health professionals who disapprove its use. Correspondingly, inpatients perceive the attitudes of professionals and their interaction as the most important factors concerning coercive measures (12). In order to avoid coercive measures through de-escalation strategies, health professionals need to have specialized training and be aware not only of the use of informal coercion but also of the importance of a respectful and empathetic attitude and ward climate, a positive admission process, as well as debriefing strategies after coercive measures (12).

### De-escalation

In comparison to other wards, the risk for aggressive behavior is increased in mental health units (13). In mental health departments, conflicts can arise as a result of interpersonal interactions between staff and patients and also between patients. De-escalation has been defined as the use of techniques including verbal and nonverbal communication skills aimed at defusing anger and stopping aggression (14). It is an approach for managing aggressive and violent behavior in a more humane manner and is arguably more dignified and less coercive than physical interventions. In addition, this guideline (14) highlights how medication can be used as a part of de-escalation strategies, but medication does not stand for de-escalation on its own. De-escalation also involves the use of verbal and physical expressions of empathy, creating therapeutic alliance, and nonconfrontational limit setting that is based on respect. The pivotal strategies focusing on de-escalation are communication, approach, de‐escalator qualities, assessment and risk, getting help, and containment measures. Different types of aggression are met with different interventions (15).

### Models of De-escalation Strategies

There are several concepts that address aggression, but little is known about successful strategies to prevent and deescalate aggressive behavior (16). Gaynes et al. (16) found in their systematic review that if there is a risk of aggressive behavior, multimodal approaches like the “Six Core Strategies” have the potential to reduce the use of restraint and seclusion. The “Six Core Strategies” were developed and supported by the National Association of State Mental Health Program Directors in the USA (17) to prevent aggressive behavior. One of its pivotal strategies concerns the commitment of institutional management such as the Chief Executive Officer (CEO) or chief medical doctors/head nurses. Leadership is described as not only the commitment to a vision, an attitude, and a plan to reduce the use of seclusion and restraint, but also the involvement of management in those practices (17). The second strategy is the use of data to inform practice, which means the monitoring of units’ or shifts’ rate of seclusion and restraint and of patients’ characteristics. The third strategy focuses on development and training of the teams toward a recovery-based treatment environment. The training involves, among other things, the exploring of rules. The authors claim—as mentioned above—that closed wards often have historic rules and procedures that are no longer appropriate to state-of-the-art treatment and not in line with a recovery-oriented, least restrictive practice (17). The fourth strategy concerns the use of prevention and assessments tools, and the fifth strategy concerns the inclusion of the patients themselves in improvement strategies or facility committees. Moreover, the inclusion of family members or peers is recommended. The last strategy focuses on debriefing techniques that aim to reduce the traumatizing effects of coercive measures for both patients and staff. Detailed, recommended questions units can ask themselves, exploring potential triggers, are, for example, “was the individual worried about anything?” or “did the individual have to wait an inordinate time for something he or she wanted?” (18). Steps for debriefing and procedures are explained and templates are available.

In Germany and Switzerland, one concept is the ProDeMa^®^ that provides a practical guideline for healthcare professionals to deal with aggression (19). The guideline aims to convey de-escalation interventions and to develop a professional approach. As in the “Six Core Strategies,” there are different steps of de-escalation. The first step, the prevention of aggression, involves getting in contact with or gaining the attention of a certain person. ProDeMa^®^ emphasizes that without contact, de-escalation may not occur. Getting in contact is linked to asking about the wishes and needs of the person. The second stage intends to change one’s own perspectives of aggressive behavior before reacting, while during the third phase, an understanding of the causes is developed. The art is not to ask “why” but, for example, “what would help.” The next two steps deal with verbal and nonverbal de-escalation techniques to calm down a person and to master a difficult situation. Nonverbal de-escalation, for example, comprises the protection of the own person. The last stage describes least restrictive and patient-friendly holding techniques, immobilization, or, in some hospitals, fixation.

### Nursing Experiences

Nursing practice is often characterized by relatively close physical contact for extended time periods, sometimes lasting over hours. Also, due to this, nurses in the psychiatric setting may be familiar with aggression toward them (20). It is known that targeted aggressive behavior can lead to anger, and (unreflected) anger can lead to reactions, which are disciplinary or even coercive (21). A strategy of de-escalation with commonly shared procedures, as the two models described before, can support balanced alternative reactions of nurses and other health professionals in a treatment team. That is why we recommend multimodal de-escalation strategies encompassing several different approaches.

## Discussion

The perception of coercion has been shown to never be neutral, but either positive or negative (22). Coercion can only be regarded as necessary when it immediately ensures integrity, autonomy, and safety of patients and staff. However, informal coercion can be seen as coercive in the sense that it still restricts patients’ voluntary and autonomous decisions. If the patient has a positive therapeutic relationship to the professional performing the coercion, he more readily perceives the coercion itself as morally right and accepts more pressure than if a stranger performed it (22). It is easier to “take advice” from someone you trust.

Does informal coercion impede the establishment of a therapeutic relationship? Or is persuasion or inducement one of these creative ideas to replace other de-escalation methods? The extent and the impact of applied informal coercion in therapeutic communication are often not recognized by practitioners, although they might interfere with a positive therapeutic relationship (6). Informal coercion is a frequently used form of communication to influence treatment outcomes. As a weaker form of coercion, it can be de-escalating if applied critically in a recovery-based environment. It needs to aim at reducing the use of seclusion and restraint and always requires moral justification and evaluation.

Common to all theories of de-escalation is the prevention of aggression by intervening before it occurs and by calming the patient. The dominant controlling attitude to calming the patient in traditional understanding should be transformed into a collaborative endeavor, where individuals are encouraged to help themselves calm down by applying their own abilities and power (23). This requires a culture of empowerment of individuals. Such a culture should also involve the critical reflection of historically evolved rules, such as groups, which are not allowed to leave, or visiting hours, which could be individually arranged. In this context, practicing de-escalation for calming the psychiatric patient may also serve as an experimental learning opportunity for patient and staff. We propose a structured and commonly shared approach encompassing critical evaluations of historic courses of action, reflections, and discussions on personal experiences and attitude, and the use of informal coercion in order to facilitate the prevention and management of aggression and violence.

## Conclusions

The spontaneous response to an aggressive behavior depends on how it was perceived, experienced, and interpreted, and depends on the attitudes and values of the perceiving person itself.

The aim of mental health practice should be to develop a high and critical awareness toward the use of coercive measures including informal coercion.

The decision to use, as well as the consideration of the least coercive measure, is an ongoing intersubjective process.

## Author Contributions

FR, CC, and RS contributed equally to the perspectives.

## Conflict of Interest Statement

The authors declare that the research was conducted in the absence of any commercial or financial relationships that could be construed as a potential conflict of interest.
